# Efficient Liposome Loading onto Surface of Mesenchymal Stem Cells via Electrostatic Interactions for Tumor-Targeted Drug Delivery

**DOI:** 10.3390/biomedicines11020558

**Published:** 2023-02-14

**Authors:** Yusuke Kono, Renpei Kamino, Soma Hirabayashi, Takuya Kishimoto, Himi Kanbara, Saki Danjo, Mika Hosokawa, Ken-ichi Ogawara

**Affiliations:** Laboratory of Pharmaceutics, Kobe Pharmaceutical University, 4-19-1 Motoyamakita-machi, Higashinada-ku, Kobe 658-8558, Japan

**Keywords:** mesenchymal stem cell, drug delivery, tumor targeting, electrostatic interaction, magnetic liposome

## Abstract

Mesenchymal stem cells (MSCs) have a tumor-homing capacity; therefore, MSCs are a promising drug delivery carrier for cancer therapy. To maintain the viability and activity of MSCs, anti-cancer drugs are preferably loaded on the surface of MSCs, rather than directly introduced into MSCs. In this study, we attempted to load liposomes on the surface of MSCs by using the magnetic anionic liposome/atelocollagen complexes that we previously developed and assessed the characters of liposome-loaded MSCs as drug carriers. We observed that large-sized magnetic anionic liposome/atelocollagen complexes were abundantly associated with MSCs via electrostatic interactions under a magnetic field, and its cellular internalization was lower than that of the small-sized complexes. Moreover, the complexes with higher atelocollagen concentrations showed lower cellular internalization than the complexes with lower atelocollagen concentrations. Based on these results, we succeeded in the efficient loading of liposomes on the surface of MSCs by using large-sized magnetic anionic liposomes complexed with a high concentration of atelocollagen. The constructed liposome-loaded MSCs showed a comparable proliferation rate and differentiation potential to non-loaded MSCs. Furthermore, the liposome-loaded MSCs efficiently adhered to vascular endothelial cells and migrated toward the conditioned medium from cancer cells in vitro and solid tumor tissue in vivo. These findings suggest that liposome-loaded MSCs could serve as an efficient cell-based drug carrier for tumor-targeted delivery.

## 1. Introduction

Nanoparticulate drug delivery carriers, such as liposomes, lipid nanoparticles, and polymeric micelles, are known to selectively accumulate in solid tumors and are, therefore, extensively exploited for improving the delivery efficiency of anti-cancer drugs to tumors and reducing their non-specific distribution in normal tissues [1,2]. Such a unique pharmacokinetic characteristic of nanoparticles largely depends on the enhanced permeability and retention (EPR) effect. However, we and other groups have reported that the delivery efficiency of nanoparticles to solid tumors is quite different between cancer types [3,4,5]. These reports demonstrated that the accumulation of nanoparticles in breast and colon tumors is much higher than that in lung, pancreatic, and melanoma tumors. This is considered to be due to the difference in not only tumor vascular permeability and density but also interstitial fluid pressures inside tumors. These findings strongly suggest that the EPR effect is not ubiquitously observed in all types of solid tumors. Therefore, the development of novel drug delivery systems targeting tumors independent of the EPR effect is strongly required.

It has been reported that several cells, such as erythrocytes, macrophages, and mesenchymal stem cells (MSCs), have the tumor-homing ability [6,7,8,9]. Therefore, these cells have attracted increasing attention as a promising cellular carrier for tumor-targeted drug delivery. Among them, MSCs have some advantages over other cellular carriers. For example, MSCs can be isolated from adult donors and easily expanded [10]. Moreover, allogeneic MSCs can be clinically used due to their poor immunogenicity [11]. For these reasons, many studies have focused on developing anti-cancer drug-loaded MSCs [8,9]. However, the direct introduction of free anti-cancer drugs into MSCs could cause cell damage and dysfunction. Moreover, MSCs express high levels of P-glycoprotein [12,13], which is responsible for not only poor intracellular loading of anti-cancer drugs but also its rapid release from MSCs. To overcome these limitations, nanoparticulate systems have been applied for more efficient drug loading into MSCs. Several studies have succeeded in the sufficient loading of anti-cancer drugs in MSCs with low cytotoxicity by using polymeric nanoparticles, and have demonstrated that these nano-engineered MSCs exhibit potent anti-tumor effect [12,14,15]. Nevertheless, there remains concerns that the introduction of anti-cancer drugs-encapsulated nanoparticles into MSCs may impair the activity and viability of MSCs via the intracellular release of their payloads. Therefore, it would be preferable if anti-cancer drug-encapsulated nanoparticles were loaded on the surface of MSCs, rather than intracellularly introduced into MSCs.

We have previously developed superparamagnetic iron oxide nanoparticle (SPION)-incorporated anionic liposome (Mag-AL)/atelocollagen (ATCOL) complexes as a safe magnetic responsive drug delivery carrier [16], and demonstrated that the complexes efficiently bound to the anionic components on the surface of MSCs via electrostatic interactions with only 30 min exposure to a magnetic field [17]. Moreover, we also observed that a large proportion of the complexes retained on the surface of MSCs at 3 h after the complexes bound to MSCs. Based on these findings, we hypothesized that the efficient loading of the liposomes on the surface of MSCs could be achieved by using Mag-AL/ATCOL complexes.

In this study, we carried out the optimization of the liposome size and composition of Mag-AL/ATCOL complexes for the efficient loading of liposomes on the surface of MSCs. Moreover, the proliferation and differentiation capacity, and the in vitro adhesive and tumor-tropic property of the constructed liposome-loaded MSCs (Lip-MSCs) were also examined.

## 2. Materials and Methods

### 2.1. Cell Culture

C57BL/6 mouse MSCs with green fluorescence protein (GFP) were purchased from Cyagen Biosciences Inc. (Santa Clara, CA, USA). The MSCs were cultured in Mouse MSC Growth Medium (Cyagen Biosciences Inc., Santa Clara, CA, USA) and used within five passages. The B16/BL6 murine melanoma cells were kindly gifted by the Cell Resource Center for Biomedical Research, Institute of Development, Aging and Cancer, Tohoku University (Sendai, Japan). The B16/BL6 cells were cultured in RPMI-1640 medium supplemented with 10% heat-inactivated fetal bovine serum (FBS), penicillin G (100 U/mL), and streptomycin (100 µg/mL). Human umbilical vein endothelial cells (HUVECs) were obtained from KURABOU (Osaka, Japan). The HUVECs were grown in HuMedia-EG2 (Kurabo Industries Ltd., Osaka, Japan). All the cells were maintained at 37 °C under 5% CO_2_/95% air.

### 2.2. Animals

Male C57BL/6N mice (6–7 weeks) were purchased from Japan SLC (Shizuoka, Japan). All the animals were maintained in a temperature-controlled animal room with free access to food and tap water. All the animal experiments were carried out in accordance with the National Institutes of Health guide for the care and use of Laboratory animals (NIH Publications No. 8023, revised 1978). The protocol was approved by the Kobe Pharmaceutical University Committee for Animal Care and Use (approval number: 2022-054). We ensured the minimization of suffering for the experimental animals.

### 2.3. Preparation of Mag-AL/ATCOL Complexes

The Mag-AL/ATCOL complexes were prepared according to our previous report [17]. For preparing Mag-AL, 1,2-distearoyl-*sn*-glycero-3-phospho-(1’-*rac*-glycerol) (NOF Inc., Tokyo, Japan), cholesterol (Nacalai Tesque, Kyoto, Japan), and DiIC18(3) (Wako Pure Chemical Industries, Ltd., Osaka, Japan) were mixed in chloroform at a molar ratio of 1:1:0.02. The lipid mixture was dried by evaporation and hydrated in a sterile Hank’s Balanced Salt Solution (HBSS, pH 6.5) containing 0.1 mg/mL of iron oxide (II, III) magnetic nanoparticles (Sigma-Aldrich, St. Louis, MO, U.S.A.) for 30 min at 75 °C under mechanical agitation. The resultant dispersion was sonicated for 30 s or 3 min using a prove-type sonicator for obtaining large-sized or small-sized Mag-AL, respectively. The particle size, ζ-potential, and polydispersity index (PDI) of the prepared Mag-AL were measured using a Zetasizer Pro (Malvern Instrument, Worcestershire, UK). The Mag-AL/ATCOL complexes were formed by gently mixing of Mag-AL with ATCOL (Koken Co., Ltd. Tokyo, Japan) at various mixing ratios, followed by incubation for 20 min at 4 °C.

### 2.4. Cellular Association and Internalization of Mag-AL/ATCOL Complexes in MSCs

The MSCs were plated in 24-well culture plates at a density of 5 × 10^4^ cells/cm^2^ and cultured for 48 h. The culture medium was removed, and 500 µL of HBSS with 5 mM glucose containing 5–200 µg lipid of Mag-AL/ATCOL complexes was added to the well. Following 3the 0 min incubation at 37 °C on a magnetic plate (OZ Biosciences, San Diego, CA, USA), the cells were washed twice with ice-cold HBSS and the cell viability was measured using Cell Counting Reagent SF (Nacalai Tesque) and a Synergy HTX multimode microplate reader (Agilent Technologies Japan, Ltd., Tokyo, Japan). Then, the cells were lysed using lysis buffer (0.5% Triton X-100, 2 mM ethylenediaminetetraacetic acid, 0.1 M Tris, pH 7.8) for measuring the total associated amount of Mag-AL. In the case of measuring the internalized Mag-AL, the cells were treated with 0.25% trypsin for 5 min, followed by incubation in 0.1% collagenase for 30 min. Then, the cells were collected using centrifugation at 250× *g* for 5 min at 4 °C, washed twice with ice-cold HBSS, and lysed using lysis buffer. The resultant lysates were centrifuged at 10,000× *g* for 10 min at 4 °C. The amount of Mag-AL in the supernatant was quantified by measuring the fluorescence intensity using a Synergy HTX multimode microplate reader.

### 2.5. Confocal Mucroscopy Study

The MSCs cultured in a 100 mm culture dish were incubated with 2 mg lipid of Mag-AL/ATCOL complexes in 10 mL HBSS with 5 mM glucose for 30 min at 37 °C on a magnetic plate. The cells were washed twice with ice-cold HBSS and collected as Lip-MSCs using a cell scraper. Then, the Lip-MSCs were seeded in a 35 mm glass bottom dish at a density of 1 × 10^4^ cells/cm^2^ and incubated for 1 h at 37 °C. The cells were washed three times with ice-cold HBSS and fixed with 4% paraformaldehyde. After washing three times with ice-cold HBSS, the cells were observed using a FLUOVIEW FV3000 confocal laser microscope (Olympus, Tokyo, Japan).

### 2.6. Cell Viability, Proliferation and Differentiation Assay of Lip-MSCs

The non-loaded MSCs or Lip-MSCs were prepared in 24-well culture plates and further incubated for 3 d. During incubation, the cell viability was measured daily using Cell Counting Reagent SF and a Synergy HTX multimode microplate reader. In addition, the non-loaded MSCs or Lip-MSCs were seeded in a 35 mm culture dish at a density of 2 × 10^4^ cells/cm^2^ and the number of cells were measured daily for 3 d.

For evaluating the differential potential of Lip-MSCs, non-loaded MSCs or Lip-MSCs were seeded in 48-well culture plates at a density of 2 × 10^4^ cells/cm^2^ and incubated for 2 d at 37 °C. Then, the culture medium was replaced with an MSC Osteogenic Differentiation Medium (Cyagen Biosciences Inc.) or MSC Adipogenic Differentiation Medium (Cyagen Biosciences Inc.) to induce osteogenic or adipogenic differentiation, respectively. The cells were incubated for 3 weeks, with the medium refreshed every 3 d. The osteogenic or adipogenic differentiation of the cells was detected using alizarin red S staining or Oil red O staining, respectively.

### 2.7. In Vitro Adhesion of Lip-MSCs to HUVECs

The HUVECs were plated in 24-well culture plates at a density of 5 × 10^4^ cells/cm^2^ and cultured until a cell monolayer was formed. Then, 5 × 10^5^ cells of the non-loaded MSCs or Lip-MSCs were added on the HUVECs monolayer and incubated for 1 h at 37 °C. After washing twice with HBSS, the adherent cells were observed using a fluorescence BZ-X810 microscope (KEYENCE Corporation, Tokyo, Japan).

### 2.8. In Vitro Migration Assay of Lip-MSCs

The cell migration assays were carried out using a 12-well cell culture insert (8 μm pore size) (Corning Life Sciences, Corning, NY, USA). The non-loaded MSCs or Lip-MSCs were suspended in serum-free medium at a concentration of 2 × 10^5^ cells/mL and 500 µL of the cell suspension was added to the upper part of the cell culture insert. The lower part of the cell culture insert was filled with 1.5 mL of RPMI-1640 medium (normal medium) with 0.5% FBS or conditioned medium collected from B16/BL6 cells with 0.5% FBS. After incubation for 24 h at 37 °C, the cells remaining at the upper surface of the membrane were removed with cotton swabs. Then, the cells that migrated to the lower surface of the membrane were fixed in 4% paraformaldehyde and observed using a fluorescence BZ-X810 microscope.

### 2.9. In Vivo Tissue Distribution Experiment

For evaluating the in vivo disposition of Lip-MSCs in the tumor and other tissues, indium-111 (^111^In)-labeled Mag-AL was prepared using our previously reported method [18]. The B16/BL6 cells (1 × 10^6^ cells suspended in 100 μL of RPMI-1640 medium) were subcutaneously inoculated into the backs of mice. When the tumor volume reached more than 300 mm^3^, ^111^In-labeled Lip-MSCs (2 × 10^6^ cells) or the corresponding amount of ^111^In-labeled Mag-AL suspended in 200 μL of phosphate-buffered saline were intravenously injected into tumor-bearing mice. The mice were sacrificed at 48 h after the administration, and the heart, lung, kidney, spleen, liver, and tumor were collected. The amount of ^111^In-labeled Mag-AL in each tissue was quantified by measuring the radioactivity using a PerkinElmer 2480 WIZARD2 automatic gamma counter (PerkinElmer Japan Co., Ltd., Kanagawa, Japan).

### 2.10. Statistical Analysis

The results are presented as the mean ± or + standard deviation (SD) of the three or four experiments. An analysis of variance (ANOVA) was used to test the statistical significance of the differences between groups. Two-group comparisons were performed using a Student’s *t*-test. Multiple comparisons between all the groups were performed using the Tukey–Kramer test.

## 3. Results

### 3.1. Optimization of the Composition of Mag-AL/ATCOL Complexes for the Efficient Loading of Liposomes on the Surface of MSCs

#### 3.1.1. Effect of Liposomes Size and ATCOL Concentration on the Cellular Association of Mag-AL/ATCOL Complexes in MSCs

**Figure 1:**
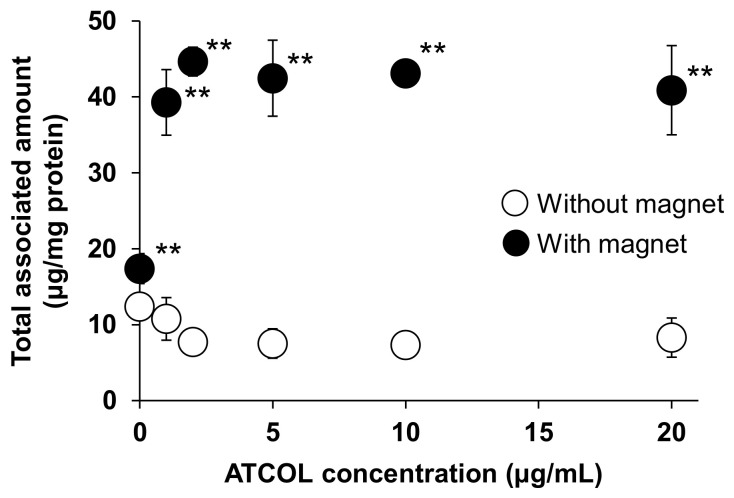
Effect of ATCOL concentrations on the cellular association and/or uptake of large-sized Mag-AL/ATCOL complexes. The complexes with a fixed concentration of large-sized Mag-AL (10 μg/mL) were added to each well and incubated for 30 min at 37 °C in the presence or absence of a magnetic field. Each value represents the mean ± SD (*n* = 4). ** *p* < 0.01, compared with the absence of a magnetic field.

We have previously reported that the cellular association of small-sized Mag-AL (particle size: 98.2 ± 4.0 nm) in MSCs was significantly increased by both the presence of a magnetic field and the formation of complexes with 1–20 µg/mL of ATCOL [17]. To evaluate the effect of the particle size of Mag-AL on the cellular association of Mag-AL/ATCOL complexes in MSCs, in the present study, large-sized Mag-AL was prepared. The particle size and ζ-potential of the large-sized Mag-AL were 594.6 ± 19.3 nm and −49.7 ± 2.7 mV, respectively (Table 1). When the large-sized Mag-AL was complexes with ATCOL, both particle size and ζ-potential were increased. We first examined the cellular association of large-sized Mag-AL/ATCOL complexes in MSCs. Similar to the results of the small-sized complexes, the large-sized Mag-AL/ATCOL complexes showed the efficient cellular association in the presence of a magnetic field (Figure 1). Moreover, the cellular associated amount of the complexes with 2–20 µg/mL of ATCOL was slightly higher than that of the complexes with 1 µg/mL of ATCOL.

**Figure 2:**
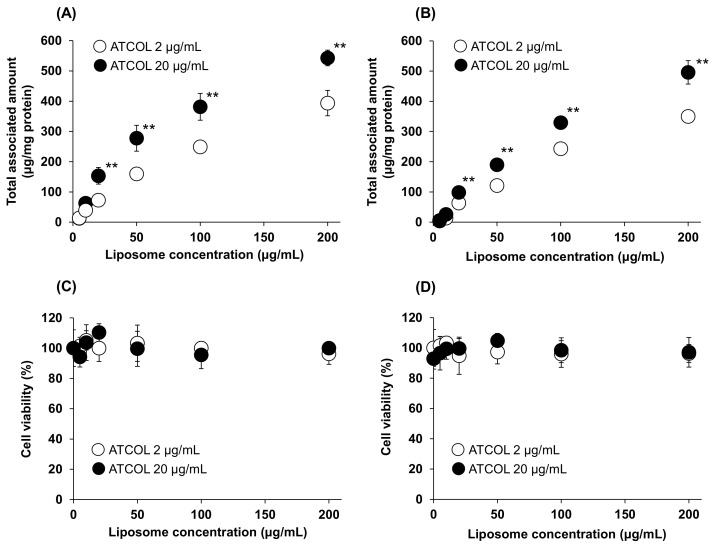
Mag-AL concentration-dependent cellular association and/or uptake of Mag-AL/ATCOL complexes and cell viability in MSCs. Small-sized (**A**,**C**) or large-sized (**B**,**D**) Mag-AL/ATCOL complexes with 2 μg/mL or 20 μg/mL of ATCOL were added to each well and incubated for 30 min at 37 °C in the presence or absence of a magnetic field. The associated amount of Mag-AL (**A**,**B**) and cell viability (**C**,**D**) were measured. Each value represents the mean ± SD (*n* = 4). ** *p* < 0.01, compared with 2 μg/mL of ATCOL.

We also investigated the Mag-AL concentration-dependent cellular association of small-sized and large-sized Mag-AL/ATCOL complexes with low (2 µg/mL) and high (20 µg/mL) concentrations of ATCOL. The cellular-associated amounts of the small-sized Mag-AL/ATCOL complexes were increased in a Mag-AL concentration-dependent manner (Figure 2A). Moreover, the small-sized complexes with 20 µg/mL of ATCOL showed a higher cellular association than the complexes with 2 µg/mL of ATCOL. Similar results were obtained with large-sized Mag-AL/ATCOL complexes (Figure 2B). However, the cellular-associated amounts of the large-sized complexes were slightly lower than those of the small-sized complexes. During these experiments, the cytotoxicity was not observed in any type of the complexes (Figure 2C,D).

#### 3.1.2. Effect of Liposomes Size and ATCOL Concentration on the Internalization of Mag-AL/ATCOL Complexes in MSCs

**Figure 3:**
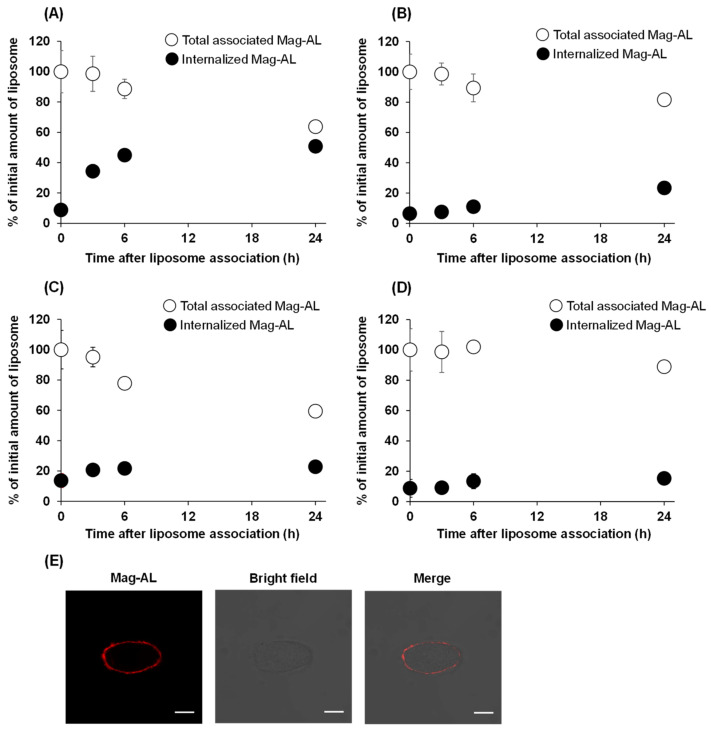
Cellular association and internalization of Mag-AL/ATCOL complexes in MSCs. Small-sized (**A**,**B**) or large-sized (**C**,**D**) Mag-AL/ATCOL complexes with 2 μg/mL (**A**,**C**) or 20 μg/mL (**B**,**D**) of ATCOL were added to each well and incubated for 30 min at 37 °C in the presence of a magnetic field. Then, the total associated or internalized amount of Mag-AL in MSCs were measured at predetermined time points. Each value represents the mean ± SD (*n* = 4). (**E**) Confocal images of MSCs loaded with large-sized Mag-AL/ATCOL complexes with 20 μg/mL of ATCOL. The complexes were added to MSCs and incubated for 30 min at 37 °C in the presence of a magnetic field. Then, the cells were collected and seeded in a glass bottom dish, followed by incubation for 1 h at 37 °C. The cells were observed using a confocal laser microscope. Scale bar: 10 μm.

Next, we evaluated the effect of the particle size and ATCOL concentration on the detachment and internalization of the Mag-AL/ATCOL complexes after the association with MSCs. As shown in Figure 3A, approximately 50% of the small-sized Mag-AL/ATCOL complexes with 2 µg/mL of ATCOL were internalized into MSCs at 24 h after the complexes were associated with the MSCs. Moreover, its total associated amount in MSCs declined to approximately 65% at 24 h, indicating that 35% of the complexes detached from the surface of the MSCs. In the case of the complexes of small-sized Mag-AL with 20 µg/mL of ATCOL, the detachment of the complexes from the cell surface was approximately 20% at 24 h after the cellular association (Figure 3B), which was lower than that of the complexes with 2 µg/mL of ATCOL. Furthermore, its internalization into MSCs was substantially suppressed to approximately 23%. Similar results about the detachment from the cell surface were observed with the large-sized Mag-AL/ATCOL complexes (Figure 3C,D). On the other hand, their cellular internalization was lower than those of the small-sized complexes. Collectively, large-sized Mag-AL/ATCOL complexes with 20 µg/mL of ATCOL showed the lowest detachment and internalization in MSCs, and the confocal laser microscopic images also demonstrated that a large proportion of the complexes were located at the edge of the cells (Figure 3E). Therefore, we decided to use these complexes for the liposome loading on the surface of the MSCs.

### 3.2. Cell Viability, Proliferation, and Differentiation Potential of Lip-MSCs

**Figure 4:**
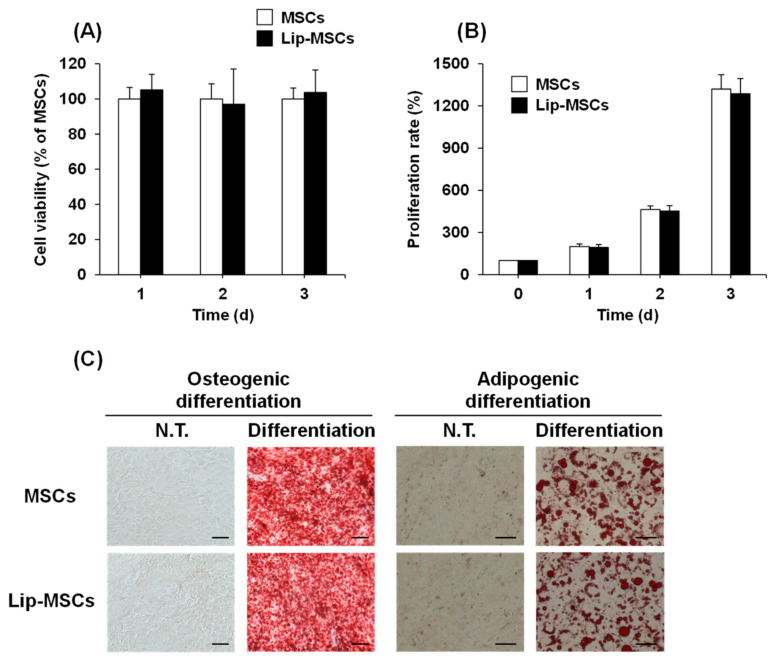
Cell viability, proliferation rate, and differentiation potential of Lip-MSCs. Non-loaded MSCs or Lip-MSCs were seeded in 24-well culture plates or 35 mm culture dishes, and cell viability (**A**) and the number of cells (**B**) were measured daily for 3 d. (**C**) The cells were incubated in osteogenic differentiation medium or adipogenic differentiation medium for 3 weeks. The osteogenic or adipogenic differentiated cells were stained using alizarin red S or Oil red O, respectively. Scale bar: 50 μm. N.T.: non-treated.

To examine whether the loading of liposomes on the surface of MSCs using Mag-AL/ATCOL complexes affects the cellular functions, the cell viability, proliferation rate, and differentiation potential of Lip-MSCs were evaluated. As shown in Figure 4A, the cell viability of the Lip-MSCs was maintained for 3 d. Moreover, the proliferation rate of the Lip-MSCs was approximately equal to that of the non-loaded MSCs (Figure 4B). In addition, the Lip-MSCs were properly differentiated into osteocytes and adipocytes after 3 weeks of culturing in each differentiation medium (Figure 4C); there was no significant difference in the degree of the osteogenic and adipogenic differentiation between non-loaded MSCs and Lip-MSCs.

### 3.3. In Vitro Adhesive and Tumor-Tropic Capacity of Lip-MSCs

**Figure 5:**
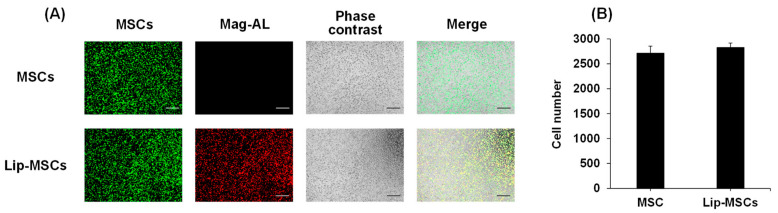
In vitro adhesion of Lip-MSCs to HUVECs monolayer. Non-loaded MSCs or Lip-MSCs were added to HUVECs monolayer cultured in 24-well culture plates, and incubated for 1 h at 37 °C. (**A**) Representative photographs of non-loaded MSCs or Lip-MSCs adhered to HUVECs monolayer. Scale bar: 500 µm. (**B**) The number of non-loaded MSCs or Lip-MSCs adhered to HUVECs monolayer. Each value represents the mean + SD (*n* = 3).

We further investigated the effect of the liposome loading on the surface of MSCs on their adhesive property to vascular endothelial cells. As shown in Figure 5, the number of adhered Lip-MCSs to the HUVECs monolayer was comparable to that of the non-loaded MSCs.

**Figure 6:**
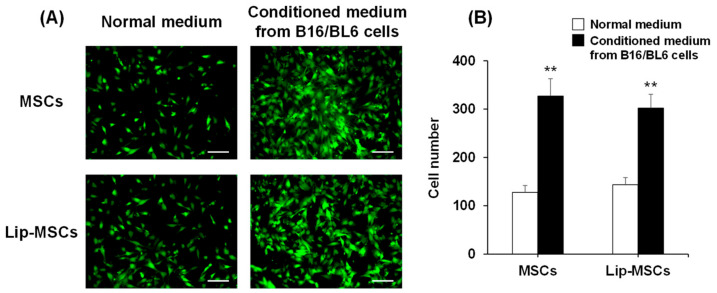
In vitro migratory activity of Lip-MSCs. (**A**) Representative photographs of non-loaded MSCs or Lip-MSCs migrated through the membrane pores toward the normal medium or conditioned medium from B16/BL6 cells. Scale bar: 100 µm. (**B**) The migrated cell number of non-loaded MSCs or Lip-MSCs. Each value represents the mean + SD (*n* = 3). ** *p* < 0.01, compared with the normal medium.

In addition, the in vitro tumor-tropic capability of the Lip-MSCs was also assessed. The number of Lip-MSCs migrated toward the conditioned medium from B16/BL6 cells was significantly higher than that toward the normal medium (Figure 6). Similar results were observed with non-loaded MSCs, and there was no statistical difference in the migrated cell number between non-loaded MSCs and Lip-MSCs.

### 3.4. In Vivo Tissue Distribution of Lip-MSCs

**Figure 7:**
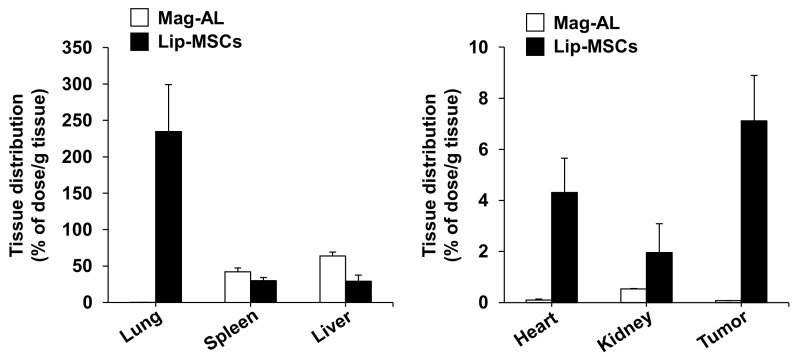
In vivo tissue distribution of Lip-MSCs after intravenous injection in tumor-bearing mice. ^111^In-labeled Lip-MSCs (2 × 10^6^ cells) or the corresponding amount of ^111^In-labeled Mag-AL were intravenously injected into B16/BL6 tumor-bearing mice, and the accumulated amount of each tissue was measured at 48 h after the administration. Each value represents the mean + SD (*n* = 4–5).

Finally, we examined the in vivo biodistribution of Lip-MSCs. When Mag-AL was intravenously injected into tumor-bearing mice, most of them were accumulated in livers (63.8 ± 5.3% of dose/g liver) and spleens (42.1 ± 5.4% of dose/g spleen) at 48 h after the administration (Figure 7). The lip-MSCs were also distributed to livers (29.2 ± 8.4% of dose/g liver) and spleens (30.0 ± 4.3% of dose/g spleen), whereas the highest accumulation was observed in lungs (234.6 ± 64.7% of dose/g lung). Moreover, Lip-MSCs exhibited a remarkable accumulation in tumors in comparison with Mag-AL (7.1 ± 1.8% of dose/g tumor vs. 0.07 ± 0.005% of dose/g tumor).

## 4. Discussions

There have been several studies on the development of surface-modified MSCs with nanoparticles. Li et al. succeeded in constructing silica nanorattle-anchored MSCs by using specific antibody-antigen recognitions on the surface of MSCs [19]. In addition, Xu et al. prepared biotinylated MSCs and biotin-modified nanoparticles, and biotin-modified nanoparticles were anchored on biotinylated the MSCs’ surfaces via biotin-avidin connection [20]. These approaches utilized chemical or biological conjugation for the cell surface modification. On the other hand, our present study exploited the electrostatic interactions for establishing simple and versatile methods for efficient liposome loading on the surface of MSCs.

Initially, we performed a comparative evaluation of the cellular association and internalization in MSCs between small-sized and large-sized Mag-AL/ATCOL complexes with low (2 µg/mL) and high (20 µg/mL) concentrations of ATCOL. With regard to the particle size, the small-sized Mag-AL/ATCOL complexes showed higher cellular association in MSCs than the large-sized complexes (Figure 2A,B). However, the small-sized complexes tend to be internalized into MSCs more than the large-sized complexes (Figure 3A,C). It has been reported that nanoparticles are mainly internalized into the MSCs through clathrin-mediated endocytosis [21,22], and the upper size limit of this pathway is approximately 200 nm [23,24]. Our results are in accordance with these previous findings. In addition to the particle size, we confirmed that the high ATCOL concentration also contributes to the lower internalization of small-sized Mag-AL/ATCOL complexes in MSCs (Figure 3A,B). Since we previously observed that the higher ATCOL concentration provided the larger particle size of the small-sized Mag-AL/ATCOL complexes [16], the suppressed endocytic uptake of the complexes with high concentrations of ATCOL would be attributed to their larger particle sizes. Furthermore, the high ATCOL concentration also suppressed the detachment of Mag-AL/ATCOL complexes from the surface of MSCs. This may be due to the stronger electrostatic interactions between the complexes and cell surfaces. Regarded together, our results demonstrate that large-sized Mag-AL/ATCOL complexes with high concentrations of ATCOL are the most suitable for the loading of liposomes on the surface of MSCs among several compositions tested because they efficiently bind to MSCs without significant detachment and internalization.

Previous studies have shown that the internalization of SPION in MSCs promotes their proliferation [25,26]. Moreover, the cellular uptake of SPIONs has also been reported to induce the osteogenic differentiation of MSCs [27]. These functional alterations of MSCs may cause the loss of their tumor-homing ability. Therefore, we examined the effect of the liposome loading on the surface of MSCs on their viability, proliferation, and differentiation potential. The cell viability, proliferation rate, and osteogenic and adipogenic differential potential of Lip-MSCs were not significantly different from those of non-loaded MSCs (Figure 4), suggesting that the activity and viability of MSCs are not impaired by the liposome loading. This would be due to the efficient binding and retention of Mag-AL/ATCOL complexes on the surface of MSCs with minimal internalization.

On the other hand, MSCs are known to express integrin α4β1 on their surface, which mediates the binding of MSCs to vascular endothelial cells [28]. Moreover, it has been reported that several chemokine receptors, such as CCR2 and CXCR4, are expressed on the surface of MSCs, and they are critically involved in the homing potential of MSCs toward tumor tissues [29,30]. Considering these background issues, there is a possibility that the liposome loading on the surface of the MSCs may mask these factors and consequently impair MSCs’ chemotaxis. Therefore, we evaluated and observed that the adhesion level of the Lip-MSCs to HUVEC was approximately equal to that of the non-loaded MSCs (Figure 5). Moreover, the in vitro migratory behavior of the Lip-MSCs toward the conditioned medium from the B16/BL6 cells was also comparable to that of the non-loaded MSCs (Figure 6). Furthermore, the in vivo accumulated amount of Lip-MSCs in tumors was approximately 100-fold larger than that of Mag-AL (Figure 7) and even approximately 1.3-fold larger than that of polyethylene glycol-modified liposomes (5.5 ± 1.1% of dose/g B16/BL6 tumor), which we previously reported [3]. These results suggest that the loading of liposomes on the surface of MSCs using Mag-AL/ATCOL complexes may hardly affect the surface properties of MSCs in terms of the tumor-homing function.

## 5. Conclusions

We succeeded in the efficient and harmless loading of liposomes on the surface of MSCs by using the complexes of large-sized Mag-AL with high concentrations of ATCOL. Moreover, we demonstrated that the prepared Lip-MSCs showed comparable in vitro adhesive and tumor-homing capacities with non-loaded MSCs. These findings suggest that Lip-MSCs could be a promising cell-based carrier for active drug targeting to solid tumors.

## Figures and Tables

**Table 1:** Physicochemical properties of magnetic anionic liposome/atelocollagen (ATCOL) complexes.

ATCOL (μg/mL)	Particle Size (nm)	ζ-Potential (mV)	Polydispersity Index (PDI)
0	594.6 ± 19.3	−49.7 ± 2.7	0.23 ± 0.02
1	604.0 ± 12.7	−38.0 ± 2.2	0.26 ± 0.04
2	761.4 ± 37.8	−35.4 ± 3.3	0.27 ± 0.06
5	817.2 ± 42.2	−24.9 ± 3.4	0.25 ± 0.02
10	1030.7 ± 54.4	−16.3 ± 3.0	0.29 ± 0.03
20	1174.4 ± 109.7	−5.1 ± 2.3	0.32 ± 0.03

Each value represents the mean ± SD (*n* = 3).

## Data Availability

All data are presented within the article.
